# Derivation of Two Critical Appraisal Scores for Trainees to Evaluate Online Educational Resources: A METRIQ Study

**DOI:** 10.5811/westjem.2016.6.30825

**Published:** 2016-07-26

**Authors:** Teresa M. Chan, Brent Thoma, Keeth Krishnan, Michelle Lin, Christopher R. Carpenter, Matt Astin, Kulamakan Kulasegaram

**Affiliations:** * McMaster University, Department of Medicine, Division of Emergency Medicine, Hamilton, Ontario, Canada; † University of Saskatchewan, Department of Emergency Medicine, Saskatoon, Saskatchewan, Canada; ‡ University of Toronto, Faculty of Medicine, Undergraduate Medical Education, Toronto, Ontario, Canada; § University of California, San Francisco, School of Medicine, Department of Emergency Medicine, San Francisco, California; ¶ Washington University School of Medicine, St. Louis, Missouri; || Mercer University School of Medicine, Department of Emergency Medicine, Department of Internal Medicine, Macon, Georgia; # University of Toronto Faculty of Medicine, Department of Family and Community Medicine, Toronto, Ontario, Canada; ** University Health Network, Department of Emergency Medicine, Wilson Centre for Health Professions Education, Toronto, Ontario, Canada

## Abstract

**Introduction:**

Online education resources (OERs), like blogs and podcasts, increasingly augment or replace traditional medical education resources such as textbooks and lectures. Trainees’ ability to evaluate these resources is poor, and few quality assessment aids have been developed to assist them. This study aimed to derive a quality evaluation instrument for this purpose.

**Methods:**

We used a three-phase methodology. In Phase 1, a previously derived list of 151 OER quality indicators was reduced to 13 items using data from published consensus-building studies (of medical educators, expert podcasters, and expert bloggers) and subsequent evaluation by our team. In Phase 2, these 13 items were converted to seven-point Likert scales used by trainee raters (n=40) to evaluate 39 OERs. The reliability and usability of these 13 rating items was determined using responses from trainee raters, and top items were used to create two OER quality evaluation instruments. In Phase 3, these instruments were compared to an external certification process (the ALiEM AIR certification) and the gestalt evaluation of the same 39 blog posts by 20 faculty educators.

**Results:**

Two quality-evaluation instruments were derived with fair inter-rater reliability: the METRIQ-8 Score (Inter class correlation coefficient [ICC]=0.30, p<0.001) and the METRIQ-5 Score (ICC=0.22, p<0.001). Both scores, when calculated using the derivation data, correlated with educator gestalt (Pearson’s r=0.35, p=0.03 and r=0.41, p<0.01, respectively) and were related to increased odds of receiving an ALiEM AIR certification (odds ratio=1.28, p=0.03; OR=1.5, p=0.004, respectively).

**Conclusion:**

Two novel scoring instruments with adequate psychometric properties were derived to assist trainees in evaluating OER quality and correlated favourably with gestalt ratings of online educational resources by faculty educators. Further testing is needed to ensure these instruments are accurate when applied by trainees.

## INTRODUCTION

With widespread access to and use of the Internet, there have increasingly been calls by the academic community for scientists to share their knowledge with the public and data with fellow researchers.^1^–^2^ Consistent with this open access movement, there has been a push to expand the repository of online educational resources (OERs). In medical education, this movement has been dubbed Free Open Access Medical education (FOAM). Social media platforms, such as blogs and podcasts, have catalyzed the proliferation of OERs partly because of their ease of publishing.^3^–^4^ Because these resources are readily accessible and literally at the fingertips of most clinicians and trainees, they are increasingly supplanting both medical journals and textbooks as a leading source of individualized, asynchronous learning.^5^–^7^ Furthermore, healthcare professionals are forming virtual communities of practice to share knowledge and network with their peers and trainees, revolving around these social media platforms.

With these new resources comes the burden of teaching learners and educators how to critically appraise them. Just as critical appraisal of primary literature is a key component of a robust medical education, so too is the ability to critically read secondary reference materials such as review papers and textbooks. However, whereas most medical school and residency curricula are required to incorporate the critical appraisal of the medical literature,^8^–^9^ little attention is given to appraising secondary resources such as textbooks, lectures, and OERs. This is concerning because inter-rater reliability of gestalt ratings of these products by trainees is quite poor.^10^ Whereas multiple critical appraisal instruments have been published to assist clinicians in the evaluation of the literature (e.g. the *Journal of the American Medical Association* User’s Guide to the Medical Literature series^11^), none have been developed for OERs.

Several recent studies have explored how to evaluate blogs and podcasts. Using a modified systematic review, Paterson et al. found 151 quality indicators for secondary resources in the existing educational literature that may be relevant for these resources.^12^ Subsequently, medical educators in various specialties as well as expert bloggers and podcasters in emergency medicine and critical care endorsed many of these quality indicators in two modified Delphi studies.^13^–^14^ Another rating tool, dubbed the *Academic Life in Emergency Medicine* Approved Instructional Resources (ALiEM AIR) Score, was developed for use by groups of medical educators.^15^ This score was based on a best approximation of what educators thought were key features of a robust blog post or podcast summary. None of these studies, however, provided a practical, simplified scoring tool to help health professionals and trainees assess the quality of OERs.

In this study, we attempted to translate the information from the previous review of the literature^12^ and modified Delphi studies^13^–^14^ to create a functional quality evaluation instrument to guide trainees in critical appraisal of blog or podcast-related written materials.

## METHODS

This study was conducted in three phases. Phase 1 reduced a previously derived and evaluated list of quality indicators to a manageable number for further assessment using data reduction techniques. Phase 2 further evaluated the remaining quality indicators in a group of trainees. We used these data to derive quality evaluation instruments and assess their reliability. Phase 3 assessed the concordance of the derived instruments with two currently accepted methods of quality evaluation (ALiEM AIR certification and educator gestalt).

An institutional review board granted an exemption for all three phases of the study. Phase 1 of the study involved the further analysis of data obtained in three previous studies^13^, ^16^–^17^ that were granted exemptions by the Hamilton Research Ethics Board (http://fhs.mcmaster.ca/healthresearch/hireb.html). Phase 2 and 3 also received an exemption. Phases 2 and 3 involved a multi-centre, web-based, cohort rating study that was conducted during April–August 2015.

### Phase 1: Quality Indicator Selection

This study built upon the work of three previously published studies. Paterson et al. defined 151 potential quality indicators that could be applied to OERs such as blogs and podcasts.^12^ This extensive list, however, is too unwieldy for learners to use practically in guiding their decision-making for appraising OERs. Subsequently, two consensus-building Delphi studies were conducted to identify what expert groups (medical educators, expert podcasters, and expert bloggers) felt were the most important quality indicators.^13^–^14^, ^18^ For the purposes of Phase 1 of this study, iterative steps were made to shorten the list of quality indicators.

**Figure 1:**
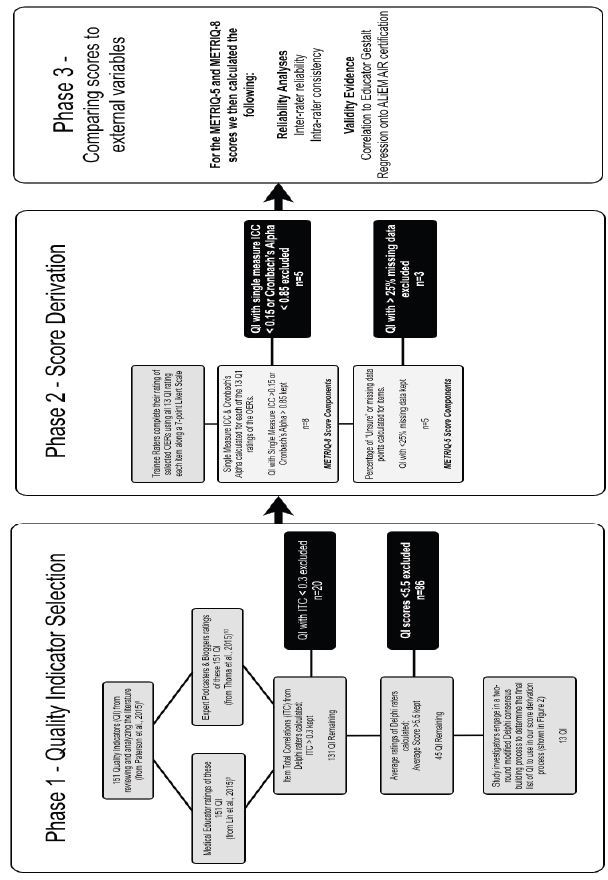
Flow diagram of study design. Phase 1 depicts the quality-indicator (QI) selection process, Phase 2 depicts the score derivation process based on the reduced list of QIs, and Phase 3 describes the reliability and validity testing data for the two derived instruments for scoring the quality of medical blogs and podcasts. *QI,* quality indicator; *ITC*, item total correlation; *ICC,* intraclass correlation coefficient; *ALiEM,* Academic Life in Emergency Medicine; *AIR,* approved instructional resources.

The overall process is depicted in Figure 1. First, we examined the priorities of expert groups (medical educators, expert OER producers) from two previous modified Delphi studies.^13^–^14^ These expert groups were selected by peer nomination via snowball sampling technique^14^ or by self-determination through attendance at an international consensus conference.^13^ In both of these studies all 151 items were ranked on seven-point Likert scales (1=strongly disagree, 7=strongly agree with item). As such, we were able to use these data to calculate item total correlations (ITC) for the 151 possible quality indicators. ITCs are an indication of the relationship between individual items and the measurement of the scale. We eliminated items with an ITC of less than 0.3, because low ITCs can be used to eliminate items that poorly fit with the scale’s measurement construct.^19^

Items with a low mean score across all the experts in the two Delphi groups (i.e. rated <5.5 on the 7-point scale) were also eliminated as possible items for our score derivation. To ensure that we valued the ratings of all groups, we also conducted a principle component analysis to look at the groupings of priorities across the groups of educators, podcasters, and bloggers.

Finally, we conducted a two-round consensus building exercise within our study team’s clinician educators (TC, BT, ML, CC, MA) to determine items we felt would be most easily rated by junior learners without training. Our team focused on eliminating items that demonstrated any of the following: required extensive knowledge or expertise, were difficult to judge without training, or were difficult to understand or define.

### Phase 2: Critical Appraisal Score Derivation

#### Rater Population and Materials

Participating collaborators were trainees (medical students, n=36; residents, n=9) from Canada and the United States, who were recruited from centers affiliated with our investigatory team and by a snowball referral process. The participants are all listed as collaborators in this study in the acknowledgments section and participated voluntarily.

The rated materials were drawn from a list of openly accessible online blog posts, previously rated for educational merit by the ALiEM AIR program ( http://www.aliem.com/new-air-series-aliem-approved-instructional-resources/).^15^ From a list of the initial 80 ALiEM AIR-rated OERs, we randomly selected 39 (20 were ALiEM AIR certified as good quality, and 19 that were not) for inclusion in Phase 2. Table 1 lists the parent websites for these 39 blog post or podcast-related OERs, and Appendix lists each OER’s website addresses and expert gestalt ratings.

#### Data Collection and OER Scoring

**Figure 2:**
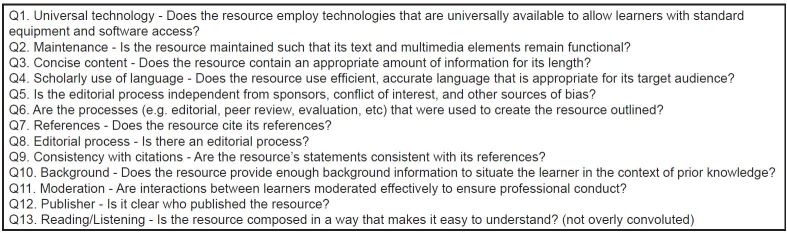
Final list of 13 quality indicators rated by trainee raters on a 7-point Likert scale.

Participating trainee raters were given three months to rate 39 OERs using a web-based Google Forms survey. Each OER was rated on 13 potential scoring system items from our reduced list (Figure 2). Each item was rated upon a seven-point Likert scale, which was anchored at 1 by the statement “Attribute not displayed,” and at 7 by the statement “Attribute displayed well.”

One OER was rated twice by each rater to allow for a calculation of intra-rater consistency. We used a modified Dillman technique to provide raters with three reminders over the study duration.^20^

#### Derivation of Our Scoring System Models

To derive our proposed scoring systems, we calculated the single measure intraclass correlation coefficient (ICC) and Cronbach’s alpha for each of the 13 potential scoring system items for all the trainee-rated OERs.^19^ We also calculated a repeated-measures ANOVA to determine intra-rater consistency for the 13 potential subscores (quality indicators) for the same rater rating the same OER at two different times.

As there were many ‘missing data’ due to rater uncertainty, we used the imputation model of substituting the grand mean for each quality indicator item to compensate for these. This imputation technique is deemed a highly conservative approach for calculating an ICC and Cronbach’s alpha. A subset of the investigatory team (TC, KK) then set a Cronbach’s alpha threshold (or average measures ICC) of ≥0.85 and a Single Measure ICC of ≥0.15 in order to derive our first scoring system model, as we felt that items that scored <0.15 in the ICC would be considered quite poor. Of note, single measure ICC measures of 0.1–0.2 are considered poor, 0.3–0.4 are considered fair, 0.5–0.6 considered moderate, 0.7–0.8 indicates strong agreement, and >0.8 indicates almost perfect.^19^ The items that met these thresholds were used to generate the first model.

Our second model incorporated the previous model, but eliminated items that generated a substantial amount of missing data (i.e. rated as “unsure”). For practicality, we felt it was important for individual raters to be able to use the quality indicator subscore items. Therefore, any items yielding a substantive amount of missing data (i.e. >25% of items were unable to be scored by the trainee raters) were eliminated as well.

### Phase 3: Comparing the scoring models with educator gestalt and ALiEM AIR ratings

#### Rater Population, Materials, and Data Collection

Participating collaborators for educator gestalt ratings were practicing academic emergency physician volunteers with a primary interest in medical education (n=20) from Canada and the United States. The participants were recruited by members of the investigatory team (TC, BT, ML, CC, MA) and are all listed as collaborators in this study in the acknowledgments section. ALiEM AIR certification status information was taken from the first six modules listed on the ALiEM.com webpage (https://www.aliem.com/aliem-approved-instructional-resources-air-series/).^14^

### Outcome Variables

#### Other Critical Appraisal Methods

Informed by the components of external validity described by Messick,^22^ we compared the scoring models to other existing measures of quality for OERs.

The 39 trainee-scored OERs were rated by educators using the same data collection method outlined in Phase 2. However, rather than rating each OER using the 13 quality indicators, the faculty were asked to use their gestalt, expert judgment to decide whether the OER would be acceptable for trainee learning. See Table 3 for the qualifications of the faculty raters. Educator’s gestalt was rated using a seven-point Likert scale (Table 2).

In addition to the educator gestalt score, the ALiEM AIR certification process served as another comparative scoring system. This was a separate rating process external to our study and raters with a separate panel of nine expert faculty panellists selecting OERs for a resident audience. The certification of these posts is openly accessible via the Internet.^21^ Of note, those who had acted as an ALiEM AIR rater were excluded from rating for this present study.

### Validity Evidence

Akin to many clinical decision rule (CDR) study designs, we opted to perform regression analyses using our two newly derived score models to determine whether they would regress to two comparative scoring instruments: the educator gestalt score and the ALiEM AIR certification using a binary logistic regression model. For the purposes of the correlation analyses, we chose to use the pragmatic score models (with substitution of a zero score when there were missing data) since individual users would not have access to grand means for the subscore components.

## RESULTS

### Phase 1: Quality Indicator Selection

The overall results and process are depicted in Figure 1. ITCs for the 151 possible quality indicators were calculated using data from the previous Delphi studies.^13^–^14^ Twenty items had an ITC<0.3, and 81 of the remaining items were rated <5.5 on the seven-point Likert scale across the two Delphi groups, and thus they were eliminated. The two-round, consensus-building exercise within our study team identified 13 of the final 45 items as being most easily rated by trainees. This list is outlined in Figure 2.

### Phase 2: Score Derivation

Table 3 depicts the demographics for the 60 total volunteers, who were recruited for the OER rating exercises.

Of this group, 28 of the 40 trainee raters (27 medical students, one resident) completely reviewed all OERs in our study. The remaining 12 trainee raters yielded incomplete datasets requiring the use of an imputation model to calculate the ICC in our score derivation procedures as described in the methods section. All 20 educators generating the gestalt ratings reviewed the complete set of OERs.

#### Intra-Rater Consistency for the 13 Quality Indicators

Since one item was rated at two different points in our rating exercise by our trainee raters, we were able to calculate a measure of internal consistency for the various items. For this analysis, we eliminated raters with incomplete data sets, using only the remaining raters to calculate a repeated-measures ANOVA to determine if there was a significant change in the quality indicator subscores when the rater encountered the OER on the second occasion. We did not detect a significant main effect of the repeated measurement occasion in our analysis (F=0.54, df (1), p=0.47). Across the 13 conditions, the first and second ratings of this item mostly correlated. We calculated the Pearson correlations for these scores, which ranged from 0.33 to 0.93 for the various items (Table 4).

#### Inter-Rater Reliability for the 13 Quality Indicators

After applying our selected imputation model (substitution of grand mean) to compensate for missing data, we calculated the intraclass correlation coefficients for each of the 13 quality indicator subscores. We used two-way random effects model for consistency measures to determine the single and average measure ICCs (Table 5). A single measure ICC allows us to understand the consistency of a randomly drawn single rater’s scores. The average measure ICC gives the reliability of the score generated by averaging or totalling the scores of *all the raters* who evaluated the OER. It can help estimate how reliability is improved by increasing the number of raters or ratings and give an indication of the actual reliability of the score generated by using several raters.^19^ This eliminated five of our possible quality indicator subscores items to generate the eight-item Score Model 1.

#### Missing Data Across the 13 Quality Indicator Subscores

Certain items yielded a high number of missing data points because participants were unsure whether to rank these items. For the purposes of deriving the score, we felt it would be prudent to generate a score model that only included items with a low number of missing data points. We therefore used a cut off of >25% missing data points within a subscore dataset to eliminate another three items from the list in Score Model 1 (eight items) to generate Score Model 2 (five items).

#### Properties of the Scores

**Figure 3:**
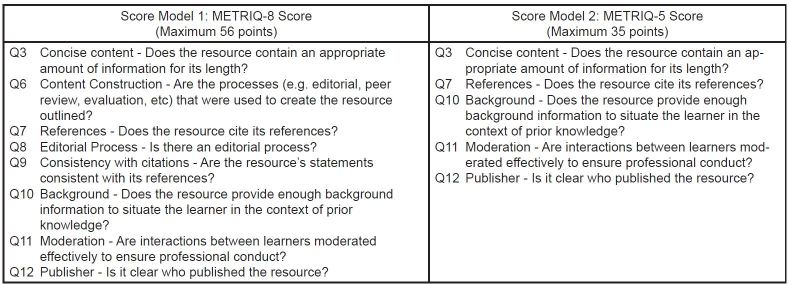
Two proposed online educational resources evaluation instruments.

Score Model 1 and 2 propose an eight-component and five-component score, respectively, which we will hereafter refer to as the METRIQ 8 Score and METRIQ 5 Score, respectively. Figure 3 lists the subscores for both OER evaluation instruments, proposed by this derivation study.

#### Reliability of the Aggregate Scores for METRIQ-8 and METRIQ-5

For the reliability calculation of the aggregate scores, we used both a pragmatic analysis which included 0-scores for any facet where a trainee rater was unsure and also an imputation analysis which included the grand mean of the subscore item. Both models were found to be moderately reliable regardless of the analytic approach with p<0.001, with the METRIQ-8 performing slightly more reliably than METRIQ-5. (Table 6).

### Phase 3: Comparing the scoring models with educator gestalt and ALiEM AIR ratings

We evaluated our scoring model instruments against both educator gestalt and ALiEM AIR certification status. We first determined the correlation between our METRIQ-8 and METRIQ-5 models and average educator gestalt score for 20 educators. We also used a logistic regression model to determine if our models would regress upon the ALiEM AIR certification status (certified or not).

#### Correlation Between Mean Educator Gestalt Score and the Average METRIQ-8 and METRIQ-5 Scores

To strengthen the validity evidence for our nascent scoring systems, we calculated the Pearson correlation statistic for the average educator gestalt scores and the pragmatic versions of both METRIQ-8 and METRIQ-5. We detected moderate correlations (p <0.05 for both) between our proposed scores and the average educator gestalt scores as shown in Table 7.

#### Logistic Regression onto ALiEM AIR Certification Status

To determine if our score had a relationship with ALiEM AIR certification, we conducted a binary logistic regression on the ALiEM AIR certification status. As demonstrated by the Wald test, this yielded a significant odds ratio for both scores. The odds ratios for METRIQ-5 and METRIQ-8 scores were 1.28, (p=0.03) and 1.5 (p=0.004) respectively.

## DISCUSSION

Teaching clinical providers the skill of critically appraisal OERs will be increasingly important as blogs and podcasts proliferate.^4^ With traditional secondary resources such as textbooks and lectures, the credibility of the source of these teachings (i.e. the editorial board of a textbook or the professorial status of a teacher) are often cited as the rationale behind why trainees and educators accept these resources as unequivocally valid without formal critical appraisal. While neither trainees nor educators have traditionally given much thought to the critical appraisal of these traditional secondary resources, the ubiquity and accessibility of OERs makes it imperative that we begin to teach trainees to be both judicious and educated in their use of these resources. Similar to what the DISCERN score did for online patient-oriented materials,^23^–^24^ our proposed METRIQ-8 and METRIQ-5 scores may allow us to ensure that trainees and educators are better able to appraise the quality of the resources they use to learn and teach, respectively.

Our investigatory team derived two scoring systems by drawing on the tradition of creating clinical decision rules (CDRs) to guide novice decision-making in patient care. We have attempted to follow a rigorous derivation process in this study, akin to those used to derive CDRs.^25^–^26^ In fact, the culmination of this study is equivalent to a Level 4 derivation study.^26^ Both of the proposed evaluation scoring instruments will require external validation. The METRIQ-8 score performs slightly better in terms of reliability. Its higher reliability may be a result of purely having more items, and thus yielding greater precision. In contrast, the METRIQ-5 score may be more easily used by trainees given its brevity (only five questions) and decreased complexity. The METRIQ-5 score may correlate better with other external measures of quality for these reasons.

Moving forward, further testing of the METRIQ scores in various populations will be required as reliability and validity are context specific, and depend on how the scores are used. METRIQ-8 and METRIQ-5 will need to be evaluated by separate and internationally diverse rater populations to provide further validity evidence, support their use, and extend their generalizability. Additionally, head-to-head comparisons with other scoring systems (such as the ALiEM AIR score, which is meant to be used by faculty members when selecting educational resources) will be necessary.^15^ We were only able to look at the relationship of our new scores with ALiEM AIR certification status (i.e. awarded or not). The use of this dichotomous data (certified or not) rather than the detailed score results (a continuous score ranging from 0 to 35) may have limited our calculations. Finally, a prospective study design looking at whether these instruments correlate with usage (i.e. webpage views or social media sharing) may be useful.

In a previous study by our research group, we found that trainees were able to select resources with single-measure ICCs of 0.22 for each other.^10^ The use of the pragmatic METRIQ-8 score improves upon this while the METRIQ-5 score approximates this consistency but further defines what may guide that gestalt. The much higher average measures ICCs suggest that a group-based rating system may be best for selection of resources for trainees. Much akin to other crowd-based rating systems (e.g. BEEM rating score^27^–^28^ and Yelp), group-based decision-making ultimately may be the best guide for rating individual resources.

## LIMITATIONS

There are several major limitations to this study. First, the use of the medical educator gestalt score as a reference standard may be questionable, since this measure has been shown to be insufficiently reliable and lacking sufficient validity evidence to provide consistent guidance to trainees.^10^ However, it is the most commonly used method for determining the quality of OERs. Second, we have used uncalibrated raters. Previous research has shown that rater cognition improves significantly if we use calibration processes such as rater-training.^29^ Third, we used a convenience sampling of raters in both the trainee and medical educator groups, which may have been biased by their contact with our investigatory group, although we attempted to sample broadly from multiple centres. We are actually quite hopeful that with rater training and calibration the use of the METRIQ scores could be improved. Fourth, our methods may be critiqued for being overly complicated. We have attempted to use robust and reproducible methods for reducing the 151 possible quality indicators that were previously found in the literature.^12^ In an effort to aggressively reduce this list, we used fairly novel methods to create two sensibly compact evaluation instruments that may be reliably applied by trainees. As such, it is prudent to compare our new scores directly with other known scores such as the ALiEM AIR before extensive use. Moreover, this study also attempts to gather some validity evidence to support the two proposed scores, but is limited because we used the non-blinded ALiEM AIR certification status of OERs to compare with our two proposed scoring instruments. Finally, many of the authors for this paper are website editors, authors, or affiliated in some way with the various blogs listed used for this study. To minimize the effects of our bias, we sought collaborators with fewer stakes and affiliations (i.e. the peer-nominated experts) to review the materials. We also included members of the team (CC, KK, KK) who are not significantly invested in these OER outlets to provide some level of objectivity and reflexivity to our investigator team.

## CONCLUSION

We have derived two possible evaluation instruments (METRIQ-8 and METRIQ-5), which may help trainees identify higher quality OERs, establish a precedent for reviewing and critically appraising secondary resources, and guide OER producers (bloggers and podcasters) to improve the quality of their educational content. These instruments correlated favourably with experienced faculty educator gestalt ratings of online educational resources.

## Supplementary Information



## Figures and Tables

**Table 1:** Parent websites and distribution of the 39 selected blog or podcast online educational resources (OER), from which the gestalt score was derived (Phase 2).

Website name	Number of rated posts
Academic Life in Emergency Medicine	12
BoringEM	1
Clinical Monster	1
Dr. Smith’s ECG blog	2
Don’t Forget The Bubbles	2
Emergency Medicine Ireland	1
EM Lyceum	3
EM Basic	1
EMCrit	1
EM Literature of Note	1
ERCast	3
Life in the Fast Lane	2
Pediatric EM Morsels	4
R.E.B.E.L EM	1
The NNT	1
The Poison Review	2
The Skeptics Guide to Emergency Medicine	1

NB: For a complete listing of all the rated blog posts, please refer to Appendix.

**Table 2:** Educator gestalt rating scale of blogs and podcasts for trainee learning.

Would you recommend this to a learner?							
0 Unsure	1 No, this is an inappropriate resource for this audience	2	3	4 This may be useful to this audience	5	6	7 Yes, this is a great resource for this audience

**Table 3:** Demographics of raters who evaluated online educational resources.

	Instrument development trainee raters (n=40)		Expert gestalt educator raters (n=20)	
% by country of origin	2.5% United States of America		75% United States of America	
	97.5% Canada		25% Canada	
Year of training or years in practice at the time of their enrollment	0 years in practice (All are trainees)		10.3 years in practice (SD 10.2)	
Academic affiliation	Year 1 medical student	40%	Full professor	10%
	Year 2 medical student	30%	Associate professor	15%
	Year 3 medical student	18%	Assistant professor	65%
	Year 4 medical student	3%	Clinical appointment	10%
	Year 1 resident	5%	None	5%
	Year 2 resident	3%		
	Year 3 resident	3%		
% current or past official medical education position within institution	N/A		90% total	
			Breakdown	
			Dean/chair	15%
			Residency PD	40%
			Residency APD	45%
			Other GME role	30%
			Clerkship director/UGME role	30%
			Research/quality Improvement role	20%

*PD,* program director; *APD*, associate or assistant program director; *GME,* graduate medical education; *UGME,* undergraduate medical education

**Table 4:** Correlations between the scores by subjects in the first and second rating incidence.

Question item number	Pearson’s *r* between the first rating and second rating of each possible quality indicator subscore item	p-value
Q1	0.92	<0.001
Q2	0.84	<0.001
Q3	0.37	0.05
Q4	0.63	<0.001
Q5	0.33	0.08
Q6	0.45	0.02
Q7	0.93	<0.001
Q8	0.57	0.001
Q9	0.74	<0.001
Q10	0.71	<0.001
Q11	0.79	<0.001
Q12	0.81	<0.001
Q13	0.85	<0.001

**Table 5:** Inter-rater agreement on the quality indicator subscore components, calculated using a 2-way random effects model for consistency to calculate the ICCs (interclass correlation coefficient).

Question item number	Single measure ICC*** (95% CI)	Average measure ICC*** (95% CI)	Number of missing data points	% Missing
Q1*	0.04 (0.02–0.08)	0.64 (0.47–0.79)	202	13%
Q2*	0.03 (0.01–0.07)	0.56 (0.35–0.74)	193	12%
Q3	0.17 (0.12–0.26)	0.89 (0.84–0.94)	206	13%
Q4*	0.12 (0.07–0.19)	0.84 (0.76–0.90)	208	13%
Q5*	0.10 (0.06–0.16)	0.81 (0.71–0.89)	713	45%
Q6**	0.28 (0.20–0.39)	0.94 (0.91–0.96)	476	30%
Q7	0.38 (0.28–0.50)	0.96 (0.94–0.98)	216	14%
Q8**	0.22 (0.15–0.32)	0.92 (0.89–0.95)	773	48%
Q9**	0.16 (0.11–0.25)	0.88 (0.82–0.93)	465	29%
Q10	0.22 (0.14–0.32)	0.92 (0.87–0.95)	287	18%
Q11	0.17 (0.11–0.26)	0.89 (0.83–0.93)	290	18%
Q12	0.29 (0.21–0.41)	0.95 (0.92–0.97)	319	20%
Q13*	0.14 (0.09–0.22)	0.87 (0.80–0.92)	285	18%

* Eliminated in Score Models 1 and 2 due to alpha <0.85 or single measure ICC <0.15

** Eliminated in Score Model 2 since trainees were unsure too often (>25% missing data)

*** p-value was <0.001 for all ICC calculated

**Table 6:** A comparison of the reliability calculations of the two proposed online educational resources evaluation instruments using different missing data procedures.

	METRIQ-8 score		METRIQ-5 score	
		
	Pragmatic analysis	Imputation analysis	Pragmatic analysis	Imputation analysis
		
Single measure ICC (95% CI)	0.30 (0.22–0.42)	0.38 (0.29–0.51)	0.22 (0.15–0.32)	0.35 (0.26–0.47)
Average measure ICC (95% CI)	0.94 (0.92–0.97)	0.96 (0.94–0.98)	0.92 (0.88–0.95)	0.96 (0.93–0.97)

*ICC,* intraclass correlation coefficient

* NB: The pragmatic analysis awards a zero value to any missing data points. The imputation analysis substitutes the grand mean for the missing data points (any items which were not rated by the trainee raters).

**Table 7:** Relationships between average METRIQ-8 and METRIQ-5 Scores with other comparative instruments (average educator gestalt score, ALiEM AIR certification).

	METRIQ-8 score pragmatic score	METRIQ-5 score pragmatic score
Pearson correlation (r) to educator gestalt score for recommending resource to a trainee	r=0.35	r=0.41
	p=0.03	p<0.01
Logistic regression for ALiEM AIR certification status	Odds ratio 1.28 (1.09–1.50)	OR = 1.5 (1.14–2.20)
	Wald test	Wald test
	(1,38)=8.8	(1,38)=8.4
	p=0.003	p=0.004
