# Supplementary material for: Derivation of Two Critical Appraisal Scores for Trainees to Evaluate Online Educational Resources: A METRIQ Study
Source: West J Emerg Med. 2016 Jul 26;17(5):574–84. doi: 10.5811/westjem.2016.6.30825 (PMC5017842; doi:10.5811/westjem.2016.6.30825)
Supplement: Supplementary file 1 [file wjem-17-574-s001.docx]

**Appendix.**

Selected ALiEM AIR-rated online educational resources and their mean expert gestalt scores for trainees (maximum score of 7) in the METRIQ-8 and METRIQ-5 derivation study (phase 2).

ALiEM AIR Certified Resources

| Parent Website | Web Address |  | Mean Expert Gestalt Score (SD)  *Max score 7* |
| --- | --- | --- | --- |
| Academic Life in Emergency Medicine | <http://www.aliem.com/approach-difficult-vascular-access/> | | 6.0 (1.07) |
|  | http://www.aliem.com/paucis-verbis-neutropenic-fever-in-cancer-patients/ | | 6.05 (0.89) |
|  | <http://www.aliem.com/pv-card-local-anesthetic-toxicity-calculations/> | | 5.00 (1.21) |
|  | <http://www.aliem.com/are-acetaminophen-levels-necessary-in-all-overdose-patients/> | | 5.70 (1.22) |
|  | <http://www.aliem.com/deep-vein-thrombosis-dvt-wells-criteria-d-dimers-happy-together/> | | 5.15 (1.07) |
|  | <http://www.aliem.com/pv-card-diagnosis-of-dvt-accp-guidelines/> | | 5.35 (1.26) |
|  | <http://www.aliem.com/dive-dive/> | | 5.65 (0.99) |
| BoringEM | http://boringem.org/2014/09/08/boring-question-med-long-qt/ | | 5.21 (1.07) |
| Clinical Monster | <http://blog.clinicalmonster.com/2013/10/why-are-we-repeating-an-already-negative-lower-extremity-doppler/> | | 5.10 (1.39) |
| EMCrit | <http://emcrit.org/podcasts/tricyclic-antidepressant-overdose/> | | 5.55 (1.04) |
| Emergency Medicine Ireland | <http://emergencymedicineireland.com/2014/02/methadone-ed/> | | 4.44 (1.33) |
| EM Lyceum | <http://emlyceum.com/2014/03/31/abscess-answers/> | | 5.45 (1.82) |
|  | <http://emlyceum.com/2014/07/01/eye-emergencies-answers/> | | 5.55 (1.50) |
|  | <http://emlyceum.com/2013/10/12/epistaxis-answers/> | | 5.85 (0.99) |
| EM Literature of Note | <http://www.emlitofnote.com/2014/02/droperidol-never-killed-anyone.html> | | 3.50 (1.27) |
| ER Cast | <http://blog.ercast.org/rivaroxaban-xarelto-for-dvt/> | | 5.90 (0.99) |
| Life in the Fast Lane | \| <http://lifeinthefastlane.com/ophthalmology-befuddler-030/> \| \| --- \| | | 6.30 (0.87) |
|  | <http://lifeinthefastlane.com/the-red-eye-challenge/> | | 5.80 (0.95) |

Other Rated Resources

| Parent Website | Web Address |  | Mean Expert Gestalt Score (SD)  *Max score 7* |
| --- | --- | --- | --- |
| Academic Life in Emergency Medicine | \| [http://www.aliem.com/trick-of-the-trade-use-the-angiocatheter- for-central-lines/](http://www.aliem.com/trick-of-the-trade-use-the-angiocatheter-for-central-lines/) \| \| --- \| | | 4.78 (1.36) |
|  | \| <http://www.aliem.com/diagnosing-hyperthyroidism/> \| \| --- \| | | 5.30 (1.12) |
|  | \| [http://www.aliem.com/trick-of-the-trade-the-pipp-for-deep-peripheral-iv- obese-patients/](http://www.aliem.com/trick-of-the-trade-the-pipp-for-deep-peripheral-iv-obese-patients/) \| \| --- \| | | 4.75 (1.17) |
|  | \| <http://www.aliem.com/thyroid-storm-treatment-strategies/> \| \| --- \| | | 6.2 (0.67) |
|  | <http://www.aliem.com/antidiabetic-medications-hypoglycemic-potential/> | | 5.05 (1.38) |
| Don't Forget the Bubbles | <http://dontforgetthebubbles.com/diabetes-insipidus/> | | 5.55 (1.04) |
|  | http://dontforgetthebubbles.com/peri-orbital-v-orbital-cellulitis/ | | 5.50 (1.14) |
| EM Basic | \| [http://embasic.org/wp-content/uploads/2014/06/39_acetaminophen- overdose-show-notes.pdf](http://embasic.org/wp-content/uploads/2014/06/39_acetaminophen-overdose-show-notes.pdf) \| \| --- \| | | 5.00 (1.47) |
| ER Cast | http://blog.ercast.org/art-chemical-takedown/ | | 4.65 (1.82) |
|  | <http://blog.ercast.org/traapped-silo-safe/> | | 4.95 (1.55) |
| HQ Med Ed | <http://hqmeded-ecg.blogspot.com/search/label/Tricyclic%20antidepressant> | | 5.65 (0.93) |
|  | <http://hqmeded.com/trial-ketamine-vs-haloperidol-severe-prehospital-agitation/> | | 5.10 (1.03) |
| R.E.B.E.L. EM | <http://rebelem.com/benefit-sodium-bicarbonate-dka/> | | 5.47 (1.12) |
| Pediatric EM Morsels | <http://pedemmorsels.com/wet-purpura-and-itp/> | | 5.61 (1.13) |
|  | <http://pedemmorsels.com/cerebral-edema-diabetic-ketoacidosis/> | | 5.72 (1.12) |
|  | <http://pedemmorsels.com/penicillin-pneumonia/> | | 5.22 (1.04) |
|  | <http://pedemmorsels.com/recurrent-croup/> | | 6.00 (0.87( |
| The Poison Review | \| <http://www.thepoisonreview.com/2014/04/28/make-the-diagnosis-sherlock-7/>* \| \| --- \| | | 5.21 (1.07) |
|  | <http://www.thepoisonreview.com/2014/08/12/review-of-scorpion-envenomation/> * | | 4.35 (1.21) |
| The Skeptics’ Guide to EM | http://thesgem.com/2013/04/sgem31-shes-got-legs/ * | | 5.10 (1.17) |
